# Omics-wide quantitative B-cell infiltration analyses identify *GPR18* for human cancer prognosis with superiority over CD20

**DOI:** 10.1038/s42003-020-0964-7

**Published:** 2020-05-12

**Authors:** Yuchen Liu, Li Wang, Kwok-Wai Lo, Vivian Wai Yan Lui

**Affiliations:** 1 School of Biomedical Sciences, Faculty of Medicine, The Chinese University of Hong Kong, Hong Kong, Hong Kong SAR; 2 Department of Anatomical and Cellular Pathology and State Key Laboratory of Translational Oncology, The Chinese University of Hong Kong, Hong Kong, Hong Kong SAR

**Keywords:** Data mining, High-throughput screening

## Abstract

Tumor-infiltrating B lymphocyte (TIL-B), and TIL-B-related biomarkers have clinical prognostic values for human cancers. CD20 (encoded by *MS4A1*) is a widely used TIL-B biomarker. Using TCGA-quantitative multiomics datasets, we first cross-compare prognostic powers of intratumoral CD20 protein, mRNA and TIL-B levels in pan-cancers. Here, we show that *MS4A1* and TIL-B are consistently prognostic in 5 cancers (head and neck, lung, cervical, kidney and low-grade glioma), while unexpectedly, CD20 protein levels lack quantitative correlations with *MS4A1*/TIL-B levels and demonstrate limited prognosticity. Subsequent bioinformatics discovery for TIL-B prognostic gene identifies a single gene, *GPR18* with stand-alone prognosticity across 9 cancers (superior over CD20), with further validations in multiple non-TCGA cohorts. *GPR18*'*s* immune signature denotes major B-cell-T-cell interactions, with its intratumoral expression strongly tied to a “T-cell active”, likely cytolytic, status across human cancers, suggesting its functional link to cytolytic T-cell activity in cancer. *GPR18* merits biological and clinical utility assessments over CD20.

## Abstract (web summary)

Yuchen Liu et al. show that GPR18 serves as a superior prognostic marker across nine cancers over CD20 by TIL-B omics analyses. GPR18 expressions in tumors are strongly correlated with major T-cell functionality scores, suggesting its functional link to cytolytic T-cell activity in cancer.

## Introduction

Tumor-infiltrating lymphocytes (TILs) are known to contribute to cancer progression, therapy responses, and patient outcomes^1,2^. Unlike the well-established antitumor roles of tumor-infiltrating cytotoxic T lymphocytes, the biological understanding of tumor-infiltrating B lymphocytes (TIL-Bs) remains limited. As its name implies, TIL-Bs were first believed to be involved in antibody-mediated immune responses (humoral responses). Yet, as of today, TIL-Bs are known to represent a complex repertoire of B-cell subtypes, comprising various B-cell subtypes [e.g., B effector 1 cells (Be1), Be2 cells, regulatory B-cells (Breg), and Killer B-cells (BK), etc.] known to exhibit a wide range of biological activities. These include antigen presentation to drive T-cell expansion, positive or negative regulations of CD4^+^T-cells, CD8^+^T-cells, regulatory T-cells and natural killer (NK) cells, as well as secretion of antitumor or pro-inflammatory cytokines (IFN-γ, IL2, TNF-α, IL10)^3,4^. It is currently believed that interactions of B-cells with other immune infiltrates, especially that with T-cells, may determine the antitumor or tumor-promoting activities of TIL-B in human cancers, possibly affecting patient outcomes^5–7^.

The B-lymphocyte antigen CD20 is encoded by the membrane-spanning 4-domains subfamily A member 1 (*MS4A1*) gene. It represents one of the most commonly used biomarkers for TIL-B thus far^8^, as CD20 protein is expressed in almost all stages of B-cell development from Pre-B-cells to memory cells, with the exception of early Pro-B and late plasma cell stages^9^. Yet, recent findings from various cancers are raising concerns regarding the use of CD20 protein alone to predict TIL-B levels in human tumors, especially for prognostic purposes. This is largely due to the widely reported obscure and inconclusive prognostic profiles of CD20^+^B-cell infiltrations across a variety of human cancers (e.g., cancers of the head and neck, lung, colon, ovary, pancreas, skin, etc.^7,10–19^), the recent discovery of CD20-negative TIL-Bs (in colorectal, breast, and ovarian cancers)^14,20,21^, and recently the complex regulations of CD20 at transcriptional, posttranslational as well as methylation levels^22,23^. This is further complicated by the semiquantitative nature of CD20 protein expression assessment (e.g., by immunohistochemistry (IHC)) and the lack of a universally defined cutoff for CD20 protein for prognostic or survival correlation studies in cancer.

In head and neck squamous cell carcinoma (HNSCC) alone, detection of CD20^+^B-cells in the past decade only revealed an obscure and inconclusive prognostic role of TIL-B for patient outcome^10,11,24^. By and large, semiquantitative detection of the single protein marker, CD20 (e.g., IHC) has generated inconclusive results with peritumoral infiltrations of B-cell in metastatic lymph nodes being associated with favorable outcome^10^, while intratumoral CD20^+^B-cell infiltration was reported to have no impact on patient outcome^11,24^. Yet, recent quantitative multigene transcriptome studies were able to consistently show specific increases of TIL-B in a subset of HNSCC tumors infected by the human papillomavirus (HPV), as well as a potentially favorable prognostic role of TIL-B in HNSCC patients in general^25–27^. In addition to HNSCC, many other cancer types also demonstrated such discrepancies for patient outcome prediction by various TIL-B detection approaches (e.g. a single CD20 marker vs. multigene TIL-B detection)^7,12–19^.

Here, based on the recent availability of highly quantitative multiomics data from the Cancer Genome Atlas (TCGA) (transcriptomic data from RNA-Seq) and the proteomic data from the Cancer Proteome Atlas (TCPA, by reverse phase protein array, RPPA) which allow quantifiable and potential functional investigations of various TILs in pan-cancers, we unbiasedly compare the prognostic powers of quantitative CD20 protein, mRNA, and TIL-B levels in 29 TCGA cancer types including HNSCC (a total of 9963 primary human tumors). Unexpectedly, Cox-regression analyses reveal limited prognosticity of quantitative CD20 protein levels vs. quantitative *MS4A1* and TIL-B levels in pan-cancers. In most cancer types, quantitative CD20 protein levels lack direct correlations with *MS4A1* or TIL-B levels, consistent with a complex regulation of CD20 expression in human tumors. Furthermore, bioinformatics attempts by TIL-B prognostic gene discovery successfully identify a single TIL-B gene, *GPR18* with its quantitative mRNA levels demonstrating stand-alone prognosticity across across cancers (superior over CD20), which is further cross-validated in independent non-TCGA cancer cohorts. *GPR18’*s immune signature denotes apparent B-cell–T-cell interactions (distinct from *MS4A1*’s “B-cell only” signature), with its intratumoral expressions tied to major cytolytic T-cell functionality scores across 28 cancers, including the cytolytic/IFN-gamma/T-effector (Teff) signature scores. *GPR18* should warrant single gene clinical utility assessments over CD20 for patient outcome prediction, as well as further biological investigations across cancers.

## Results

### CD20 mRNA and TIL-B are associated with HNSCC survival

Taking advantage of the recent availability of quantitative proteomics, transcriptomic, and survival data of TCGA-HNSCC cohort, we unbiasedly cross-compared the prognostic values of intratumoral CD20 protein, CD20 mRNA (encoded by the gene *MS4A1*), and TIL-B levels by univariate Cox-regression analyses. Quantitative CD20 protein data of the TCPA (level 4 normalized RPPA data, October 2019), and quantitative *CD20* mRNA (*MS4A1*) RNA-Seq expression data from TCGA were used. TIL-B levels were computed using the multigene bioinformatics approach, tumor immune estimation resources (TIMER)^25^, which has been successfully cross-validated in multiple cancer types^28,29^. In HNSCC, TIMER-computed infiltration levels of six immune cell types, including TIL-B, used a total of 449 immune marker genes with removal of outlier genes to avoid quantitative bias during computation^25^ (Supplementary Table 1). *MS4A1* was defined as an outlier by TIMER due to its extreme high expressions in B-cell lines, and thus excluded during TIL-B calculations (Supplementary Fig. 1). Therefore, TIMER-based TIL-B levels will not exhibit any weighing bias toward CD20-positive vs. CD20-negative TIL-B in a tumor.

**Fig. 1:**
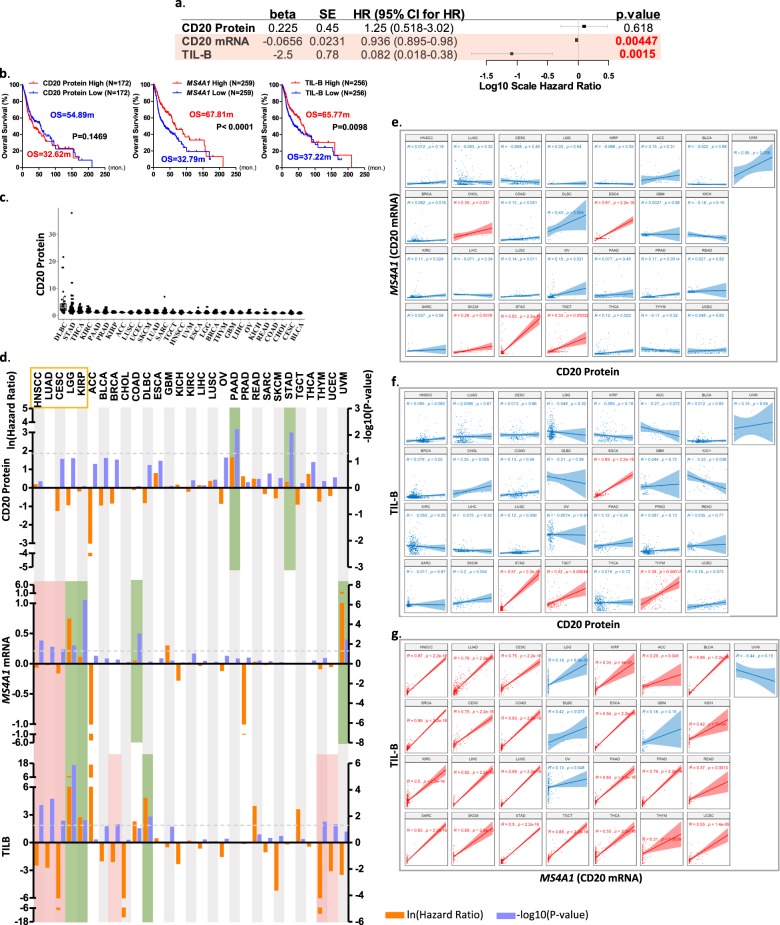
Quantitative levels of TIL-B and *MS4A1*, but not CD20 protein, are consistently prognostic for HNSCC, LUAD, CESC, LGG, and KIRP. **a** A forest plot showing the univariate Cox-regression analyses for quantitative CD20 protein (*N* = 344), CD20 mRNA (*MS4A1*, *N* = 518) and TIL-B (*N* = 512) levels in HNSCC. **b** Kaplan–Meier survival curves for high and low levels of quantitative CD20 protein, *MS4A1*, TIL-B (median cutoffs) in HNSCC patients. **c** Quantitative CD20 protein levels across 29 cancer types in TCPA datasets. **d** Comparison of the prognostic powers of levels of TIL-B, *MS4A1* mRNA, and CD20 protein in 29 cancer types. Pink and green colors represent the positively and negatively prognostic for cancer types, respectively. Pearson’s correlations between CD20 protein and *MS4A1* mRNA expression levels (**e**), between CD20 protein and TIL-B levels (**f**), and between *MS4A1* mRNA and TIL-B levels (**g**) in 29 cancer types. The red plots represent positive correlations (Pearson *R* > 0.2 and *P* < 0.05) and the blue plots represent no or negative correlations. The *N* numbers for respective analyses are shown in Supplementary Table 2.

For HNSCC prognosis, Cox-regression revealed that quantitative levels of TIL-B (*P* = 0.0015, hazard ratio [HR] = 0.082) and *MS4A1* (*P* = 0.00447, HR = 0.936), but not quantitative CD20 protein levels (*P* = n.s.), were significantly associated with patients’ overall survival (OS) (Fig. 1a). Subsequent Kaplan–Meier survival analyses also revealed a lack of prognostic significance by CD20 protein level in HNSCC, as opposed to consistent and statistically significant prognosticity by *MS4A1* and TIL-B levels (Fig. 1b, median cutoffs). In general, high intratumoral *MS4A1* or TIL-B levels above median indicated improved OS vs. respective low groups (Fig. 1b). In fact, across all cutoffs examined, CD20 protein levels did not predict HNSCC patient survival (Supplementary Fig. 2). This lack of prognosticity by quantitative CD20 protein level for TCGA-HNSCC dataset was consistent with several previous reports using semiquantitative IHC scoring of CD20 protein expressions in independent HNSCC cohorts^11,24^. Yet, the single gene *MS4A1* and multigene TIL-B levels appeared to be consistently prognostic for HNSCC patient outcomes, consistent with the known abundances of activated B-cell, antigen-presenting B-cell, and memory B-cells in HNSCC patient tumors, potentially supportive of the antitumor activity of TIL-B as recently reported^30^.

### CD20 protein levels are only prognostic for pancreatic adenocarcinoma (PAAD) and stomach adenocarcinoma (STAD)

Driven by our HNSCC findings above, we sought to examine the prognosticity of CD20 proteins vs. *MS4A1* mRNA and TIL-B levels across all human cancers in the databases. Among pan-cancers, diffuse large B-cell lymphoma (DLBC) has the highest intratumoral CD20 protein level (Fig. 1c), consistent with the CD20-positive B-cell malignancy nature of DLBC^31,32^. STAD, thyroid carcinoma (THCA), and kidney renal clear cell carcinoma (KIRC) have relatively higher CD20 protein expressions vs. the remaining 25 cancer types. The *N* numbers of respective pan-cancer analyses in this study are shown in Supplementary Table 2. Strikingly, consistent with our findings in HNSCC, Cox-regression analyses also revealed the lack of prognostic power of quantitative CD20 protein levels across pan-cancers, except for two gastrointestinal tract cancers, namely PAAD and STAD. Elevated CD20 protein levels were associated with decreased OS in PAAD (*P* = 0.00605, HR = 5.22) and STAD (*P* = 0.00798, HR = 1.38; Fig. 1d, Supplementary Fig. 3). The small uveal melanoma proteomic dataset (UVM; *N* = 12) could not be reliably analyzed.

As CD20 protein is encoded by the gene *MS4A1*, expressions of them are expected to be correlated. Strikingly, we found that in as many as 24 TCGA cancer types, quantitative CD20 protein levels lacked significant correlations with *MS4A1* mRNA levels (Pearson *R* > 0.2, *P* < 0.05 as cutoffs), except for five cancer types [esophageal carcinoma (ESCA), STAD, testicular germ cell tumors (TGCT), cholangiocarcinoma (CHOL), and skin cutaneous melanoma (SKCM)] (Fig. 1e). Similarly, CD20 protein levels also lacked quantitative correlations with TIL-B in as many as 25 cancers, except for ESCA, STAD, TGCT, and thymoma (THYM) (Fig. 1f). In HNSCC, CD20 protein levels did not correlate with *MS4A1* nor TIL-B levels (*P* = n.s, Fig. 1e, f).

### TIL-B and MS4A1 display prognosticity across five cancer types

Compiling together the prognostic powers of TIL-B, *MS4A1* mRNA, and CD20 protein levels on patient outcomes across 29 TCGA cancers by Cox-regression analyses, we identified a total of five cancer types in which intratumoral *MS4A1* and TIL-B levels were consistently prognostic: TIL-B-high or *MS4A1*-high were positively prognostic for HNSCC, lung adenocarcinoma (LUAD), and cervical squamous cell carcinoma and endocervical adenocarcinoma (CESC), but negatively prognostic for brain lower grade glioma (LGG) and kidney renal papillary cell carcinoma (KIRP) (Fig. 1d). As a single gene, *MS4A1* mRNA levels alone were prognostic in seven cancers (UVM, LGG, KIRP, colon adenocarcinoma (COAD), LUAD, HNSCC, and CESC), while TIL-B levels alone were prognostic for nine cancer types (LGG, DLBC, KIRP, breast invasive carcinoma (BRCA), HNSCC, LUAD, uterine corpus endometrial carcinoma (UCEC), THYM, and CESC; Fig. 1d). Importantly, though TIMER excludes *MS4A1* gene expression for TIL-B calculation, we still observed high correlations between *MS4A1* and TIL-B levels in HNSCC (*R* = 0.87, P < 2.2e−16), and 23 additional cancers (Pearson *R* > 0.2, *P*  < 0.05) (Fig. 1g), indicating the robustness of TIMER in TIL-B computation across cancers.

### Unfavorable-prognostic cancers display elevated IL6/PD-L2

**Fig. 2:**
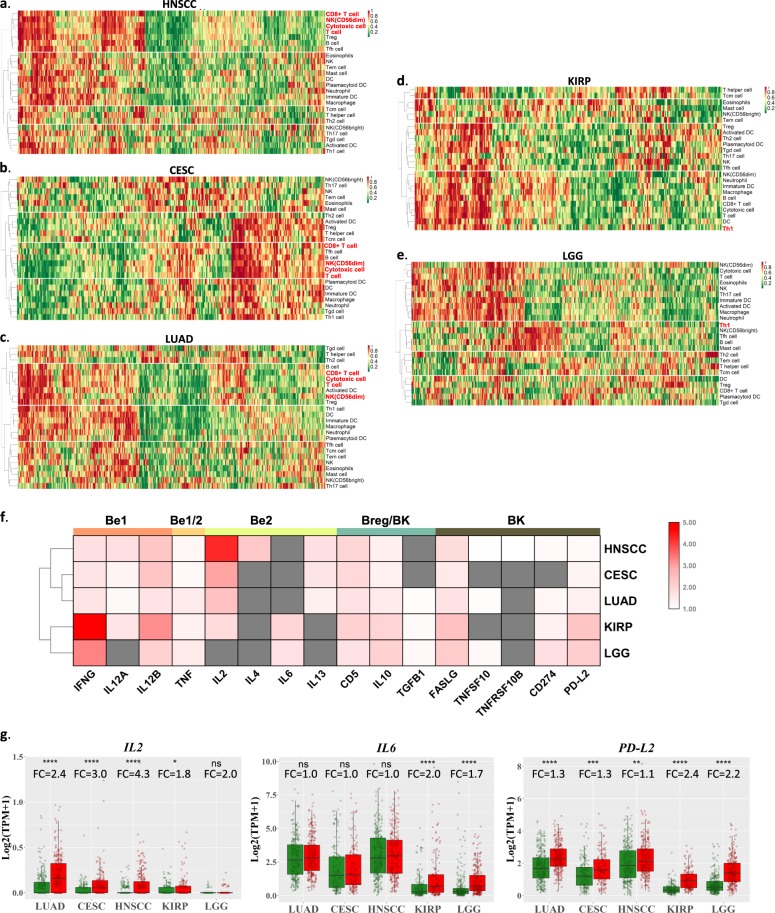
Unfavorable-prognostic cancers display TIL-B-cell cluster with Th1 and elevated IL6 and PD-L2 B-cell subtype signatures. Clustering of 24 ssGSEA-based immune cell types in HNSCC (*N* = 520) (**a**), CESC (*N* = 304) (**b**), LUAD (*N* = 515) (**c**), KIRP (*N* = 290) (**d**), and LGG (*N* = 516) (**e**). **f** Fold changes of log2 (TPM + 1) of Be1, Be2, Breg, and BK marker genes between TIL-B high and low tumors in HNSCC (*N* = 512), LUAD (*N* = 502), CESC (*N* = 304), KIRP (*N* = 289), and LGG (*N* = 514) (median cutoff). Unpaired Student’s *t* test *P* < 0.05 ones are shown in color, and the nonsignificant ones are in gray. **g** Comparison of *IL2*, *IL6*, and *PD-L2* expression levels between TIL-B high and low tumors (median cutoff) in HNSCC, LUAD, CESC, KIPR, and LGG. The boxplot elements are defined as follows: center line, median; box limits, upper and lower quantiles; points, all data point. Significance was calculated with unpaired Student’s *t* test. FC fold change, Tfh cell T follicular helper cell, Th1 type 1 T helper, Th2 type 2 T helper, Th17 T helper 17, Tcm cell central memory T-cell, Tem cell effector memory T-cell, Tgd cell gamma delta T-cell, Treg cell regulatory T-cell, DC dendritic cell, aDC activated dendritic cell, iDC immature dendritic cell, pDC plasmacytoid dendritic cell, NK natural killer.

Infiltrating B-cells can be anti- or protumorigenic, depending on their interactions with other immune cells in the tumor microenvironment and the B-cell subtypes involved. Here, using single sample gene set enrichment analysis (ssGSEA)^33^, we first determined the intratumoral levels of 24 immune cell types (including TIL-B) in these five TIL-B/*MS4A1*-prognostic cancers, followed by nonhierarchical clustering analysis to determine their relationships with TIL-B in patients’ tumors. Notably, the three favorable prognostic cancers (HNSCC/CESC/LUAD) uniformly showed TIL-B clustering with CD8^+^T-cell, cytotoxic cell, T-cell, and NK(CD56^dim^), all known to mediate cytolytic antitumor responses (Fig. 2a–c). In contrast, in both unfavorable-prognostic cancer types, LGG and KIRP, TIL-B clustered with Th1 cells, a well-known inefficient inducer of polyclonal B-cell proliferation^34^ (despite KIRP’s clustering with cytolytic cells; Fig. 2d, e). Particularly in LGG, TIL-B also clustered with NK(CD56^bright^) cells, which are ineffective antitumor responders^35^ (Fig. 2e).

Next, we examined the four major B-cell subtype signatures defined by Spaner and Bahlo^36^ (Be1, Be2, Breg, BK) in these cancers. Interestingly, the good and worse TIL-B/*MS4A1*-prognostic cancers showed remarkable differences in Be2 and BK signature genes (Fig. 2f). With regard to Be2 signature genes, the B-cell stimulatory cytokine *IL2*^37^ was the most elevated in TIL-B-high HNSCC/CESC/LUAD, while *IL6*, a pro-inflammatory signal for cancer progression and initiation of germinal center formation^38,39^, was elevated in TIL-B-high KIRP/LGG tumors (Fig. 2f, g). Importantly, for the immunosuppressive BK signature gene, *PD-L2*, TIL-B-high KIRP/LGG showed the greatest upregulations of 2.2–2.4 log2 fold increases (=3.1-fold in linear scale) as compared with only 1.1–1.3 log2 fold increases (=1.4–1.5-fold in linear scale) in TIL-B-high LUAD/HNSCC/CESC (vs. their respective low-TIL-B tumors, Fig. 2f, g). Since *PD-L2* can suppress T-cell function, our findings may indicate a negative T–B-cell interaction in LGG and KIRP patient tumors. Overall, our findings from immune cell type clustering and B-cell subtype signature analyses may help explain patient outcomes in these TIL-B/*MS4A1*-prognostic cancers.

### GPR18 is a stand-alone prognostic marker in multiple cancers

**Fig. 3:**
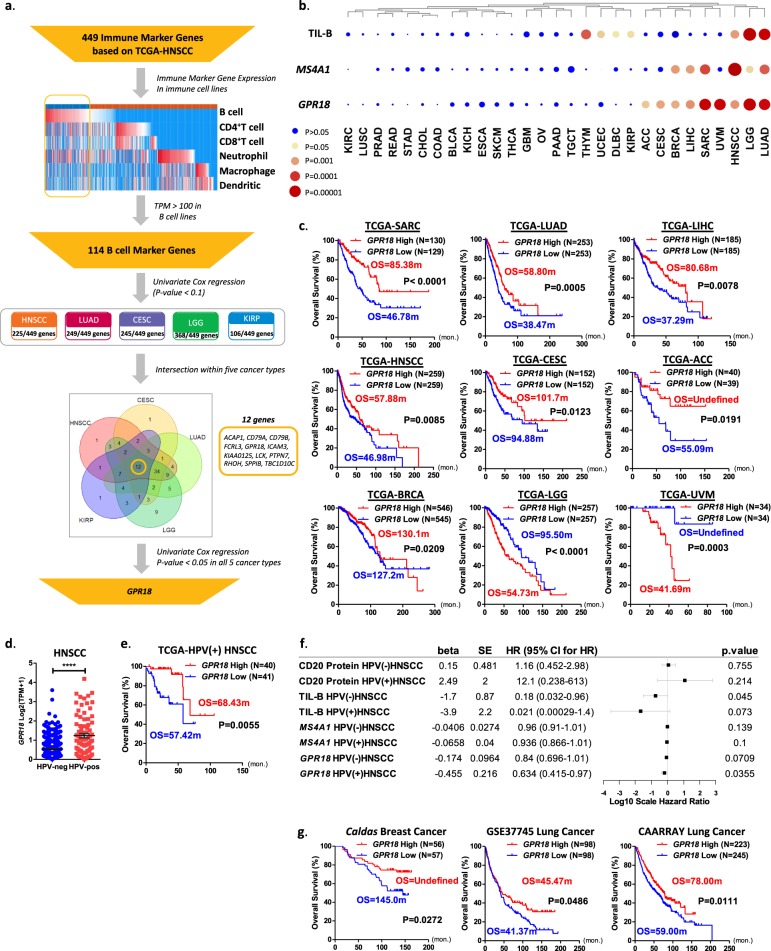
*GPR18* is a stand-alone prognostic TIL-B gene across nine cancer types. **a** Schematics for B-cell prognostic signature gene analyses in HNSCC, LUAD, CESC, LGG, and KIRP, which revealed a single gene, *GPR18*, whose mRNA expression level is prognostic across several cancer types. **b** Comparison of the significance of log-rank tests between TIL-B, *MS4A1*, and *GPR18* in 29 cancer types using median cutoff. **c** Kaplan–Meier plot demonstrating the versatility of *GPR18* mRNA expression levels for prognostication of nine cancer types with median cutoff. The *N* numbers are shown in supplementary Table 2. **d** In HNSCC, *GPR18* expression level was higher in HPV(+) tumors (*N* = 81) than HPV(−) (*N* = 416) ones. **e** Kaplan–Meier plot showing a longer OS for *GPR18-*high patients than *GPR18-*low patients in HPV(+)HNSCC (median cutoff). **f** Forest plot showing univariate Cox-regression analyses of quantitative CD20 protein, TIL-B, *MS4A1* mRNA, and *GPR18* mRNA levels in both HPV(+)HNSCC and HPV(−)HNSCC. The *N* numbers for CD20 protein, TIL-B, *MS4A1* mRNA, and *GPR18* mRNA in HPV(+)HNSCC are 42, 79, 81, and 81, respectively, and in HPV(−)HNSCC are 288, 412, 416, and 416, respectively. **g** Kaplan–Meier plot showing statistically significant prognostic power of *GPR18* mRNA levels (median cutoff) in TCGA-independent cohorts of breast and lung cancers with microarray expression data.

As *MS4A1*/TIL-B levels demonstrated prognosticity in five cancer types only, and TIMER often requires comprehensive RNA-Seq data with deconvolution computations, clinic-wide implementation of TIMER-based TIL-B prognostic evaluation could be challenging. Besides, *MS4A1* mRNA detection may also present bias toward CD20-positive TIL-B-cell detection. Therefore, we sought to identify additional TIL-B genes for prognostic purposes. We employed the TIMER TIL-B gene set for such an exploration. The informatics scheme is depicted in Fig. 3a. Among the 449 TIMER immune marker genes denoting six immune cell types in TCGA-HNSCC (Supplementary Table 1), we identified 114 genes with decent expressions in B-cell lines as TIL-B marker genes (average transcript per million (TPM) > 100; Supplementary Table 3). Cox-regression analyses were then performed for the five TIL-B/*MS4A1*-prognostic cancer types. A total of 12 genes displayed *P* values < 0.1 (*ACAP1, CD79A, CD79B, FCRL3, GPR18, ICAM3, KIAA0125, LCK, PTPN7, RHOH, SPPIB*, and *TBC1D10C*). Strikingly, further filtering with Cox-regression *P* value < 0.05 exposed a single gene named *GPR18*, whose mRNA expression levels displayed significant prognosticity across nine cancer types (log-rank test *P* < 0.05, median TPM cutoff; Fig. 3b, c). They are sarcoma (SARC), LUAD, liver hepatocellular carcinoma (LIHC), HNSCC, CESC, adrenocortical carcinoma (ACC), BRCA, LGG, and UVM.

HNSCC comprises two subtypes based on HPV-positivity. They are HPV(−)HNSCC and HPV(+)HNSCC. We found that *GPR18* mRNA expression level was markedly upregulated in HPV(+)HNSCC when compared with HPV(−)HNSCC (Fig. 3d). Furthermore, HPV(+)HNSCC patients with elevated tumoral *GPR18* expressions were found to have significantly longer OS than patients with low *GPR18* expressions (median cutoff, OS of 68.43 vs. 57.42 months, *P* = 0.0055, Fig. 3e). Importantly, the *GPR18* mRNA levels were prognostic among HPV(+)HNSCC patients (HR = 0.634, *P* = 0.0355), superior to TIL-B, *MS4A1* mRNA, and CD20 protein levels, which were not prognostic in HPV(+)HNSCC at all (Fig. 3f).

### Cross-validation of *GPR18* mRNA in independent cancer cohorts

Next, we independently examined the prognosticity of *GPR18* mRNA expressions in several non-TCGA cancer cohorts with long-term survival data (>150 months or 12 years) and microarray RNA expression data. As shown in Fig. 3g, we were able to cross-validate the prognosticity of *GPR18* mRNA expression levels (median cutoffs) in the Caldas early breast cancer cohort^40^ (*N* = 113), as well as two independent lung cancer cohorts, namely the GSE37745 (*N* = 196)^41^ and CAARRAY (*N* = 468) using Kaplan–Meier plotter^42^. For the Caldas early breast cancer cohort, *GPR18*-high patients showed much improved OS (undefined; median OS has not yet been reached) vs. 145 months. Similarly, for the CAARRAY and GSE37745 lung cohorts, *GPR18*-high expressors survived longer than *GPR18*-low expressors (Fig. 3g). Thus, *GPR18* levels appear to be prognostic in both TCGA and non-TCGA cancers.

### GPR18 indicates B-cell–T-cell interactions

**Fig. 4:**
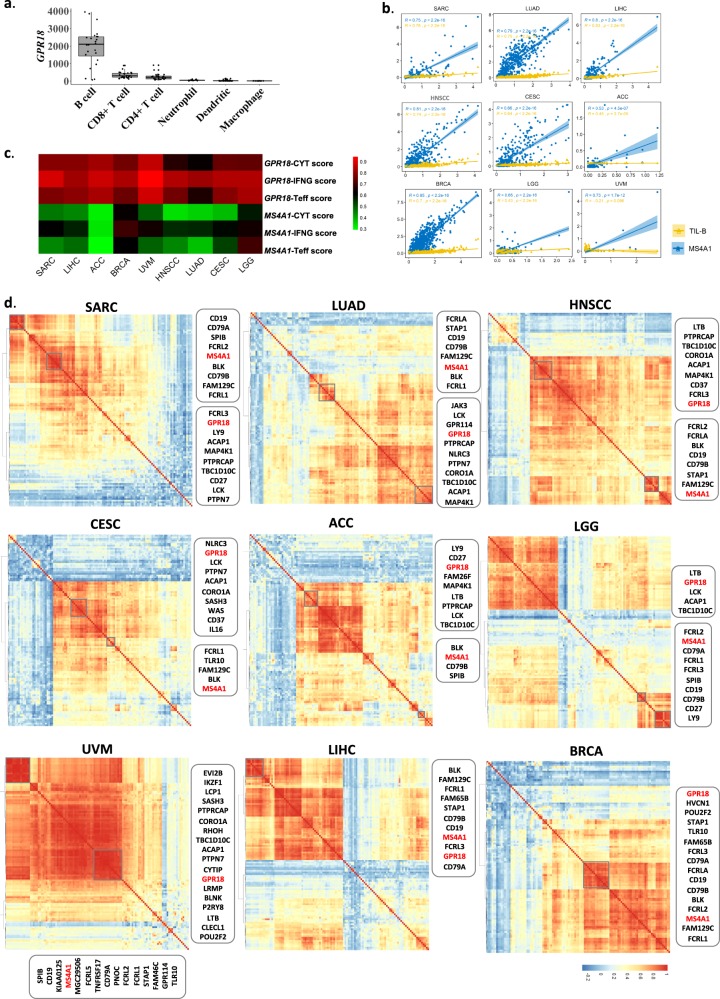
*GPR18* indicates B-cell–T-cell interaction. **a** *GPR18* expression levels (normalized values) in six TIMER-related immune cell types (B-cell, *N* = 24. CD4^+^ T-cell, *N* = 27. CD8^+^ T-cell, *N* = 28. Dendritic, *N* = 88. Macrophage, *N* = 15. Neutrophil, *N* = 18). **b** Heatmap of Pearson’s correlations between *GPR18* and *MS4A1*/TIL-B levels in nine *GPR18*-prognostic cancer types (SARC, *N* = 257. LIHC, *N* = 371. ACC, *N* = 79. BRCA, *N* = 1091. UVM, *N* = 80. HNSCC, *N* = 514. LUAD, *N* = 511. CESC, *N* = 304. LGG, *N* = 515). **c** Pearson’s correlations between *GPR18*/*MS4A1* and three T-cell immunoreactive signature scores (CYT, Teff, and IFNG) in nine *GPR18*-prognostic cancer types (SARC, *N* = 257. LIHC, *N* = 371. ACC, *N* = 79. BRCA, *N* = 1091. UVM, *N* = 80. HNSCC, *N* = 514. LUAD, *N* = 511. CESC, *N* = 304. LGG, *N* = 515). **d** Expression correlation heatmaps of 114 TIMER-based B-cell marker genes plus *MS4A1* in nine *GPR18*-prognostic cancer types (SARC, *N* = 259. LIHC, *N* = 371. ACC, *N* = 79. BRCA, *N* = 1093. UVM, *N* = 80. HNSCC, *N* = 520. LUAD, *N* = 515 CESC, *N* = 304. LGG, *N* = 516).

*GPR18* encodes the cell surface G-protein coupled receptor 18. According to the RNA-Seq expression profiles in 27 different human organs^43^, *GPR18* was found to be highly expressed in major immune organs of hematopoietic and immune lineages, including lymph nodes, spleen, bone marrow, etc. Recently, *GPR18* has been reported to play a role in the establishment of CD8 effector T-cell compartment^44^ and maintenance of normal CD8αα intraepithelial lymphocytes compartment in the small intestine^45^, suggestive of its potential functional interactions with T-cells. As of today, little is known about *GPR18*’s role in B-cell immunity, except for its high expressions in mature follicular B-cells^46^. In fact, our TIMER analysis also showed the highest *GPR18* mRNA expression in B-cells (Fig. 4a). In pan-cancers, *GPR18* mRNA expression levels demonstrated extremely significant Pearson correlations (Pearson *R* values) with *MS4A1* and TIL-B levels across all 29 cancer types, except for DLBC and UVM (*R* > 0.2, *P* < 0.0001; Fig. 4b, Supplementary Fig. 4).

To further identify the possible immune functions of *GPR18* in *GPR18*-prognostic cancers, we determined immune gene signatures that clustered with *GPR18* by non-hierarchical clustering of expression correlations of all 114 TIL-B marker genes defined by TIMER. For this particular analysis, we also included *MS4A1* in order to examine how *GPR18* was related to or different from *MS4A1* functions. Strikingly, *GPR18* was found to be almost uniformly clustered with B-cell gene sets involved in B-cell–T-cell interactions in 7/9 cancers. Whereas *MS4A1* has a “B-cell only” functional signature clustering with genes involved in B-cell receptor (BCR) signaling, B-cell proliferation and differentiation (Fig. 4d). Specifically, these B-cell–T-cell interaction genes include *ACAP1, LCK*, *PTPRCAP*, *TBC1D10C*, *CORO1A*, *LTB*, *MAP4K1*, and *PTPN7* (Fig. 4d, Supplementary Table 4). In particular, *ACAP1* participates in B-cell signaling and antigen presentation to CD8^+^T-cell^47^. *LCK* phosphorylates TCR upon its engagement with antigen-presenting cells^48^. *PTPRCAP* and *TBC1D10C* are involved in B-cell–T-cell activation and regulation^49,50^. *MAP4K1* connects TCR or BCR to SAPK/JNK and IκB kinase signaling in lymphocytes^51^. *CORO1A* is related to TCRαβ-induced signaling^52^, while *PTPN7* can attenuate T-cell activation^53^. In sum, the majority of genes co-expressed with *GPR18* were all functioning to regulate T-cell-mediated immunity.

Based on our findings and recent reports that *GPR18* was involved in regulating CD8^+^T-cell environment, we further examined if *GPR18*-high tumors would have any functional indications on T-cell activity by assessing three well-established T-cell immunoreactive functionality scores. These were the cytolytic score (CYT), Teff, and the antitumor IFNG signature score^54–56^. As shown in Fig. 4c, *GPR18* showed higher correlation coefficients with all three CYT, Teff, and IFNG scores than *MS4A1* in the nine *GPR18-*prognositc cancer types, as well as 19 additional cancer types (except for DLBC; Supplementary Fig. 5). In addition, *GPR18* is closely co-expressed with several cytotoxic CD8^+^ T-cell markers in 28/29 cancer types (Supplementary Figs. 6 and 7), indicating that *GPR18*-expressing TIL-B-cells may be specifically linked to cytotoxic T-cell functions across human cancers.

## Discussion

For the first time to our knowledge, we were able to cross-compare in an omics-wide manner the prognostication powers of quantitative levels of CD20 protein, CD20 mRNA (*MS4A1*), and TIL-B in pan-cancers using highly quantitative proteomics from TCPA and transcriptomic data from TCGA. First, we identified that quantitative levels of CD20 protein, the most widely used TIL-B marker, were only prognostic for patient OS in PAAD and STAD. Our findings are consistent with recent findings from Murakami et al. that intratumoral increase in Bregs could contribute to immune evasion in stomach cancer and high intratumoral Bregs in stomach cancer patients was associated with poorer outcome in a 5-year survival analysis^57^. Bregs are known to express CD20 and contribute to immunosuppression in cancer^58^. Thus, Breg-expressed CD20 protein may indicate poor prognosis in STAD. For pancreatic cancer, Gunderson et al. reported that pancreatic tumor growth in either B-cell-deficient mice or Ig receptor gamma null FcRγ^−/−^ mice were smaller than in littermate controls^59^, implicating a pro-tumorigenic role of B-cells in this cancer type. A subsequent study by the group suggested that B-cells, via collaboration with myeloid cells, might contribute to PAAD tumor growth.

More strikingly, across cancer types (except for ESCA, STAD, and TGCT cancers), CD20 protein levels do not correlate with *MS4A1* nor TIL-B levels. Whereas quantitative expression levels of the single gene, *MS4A1* (CD20 mRNA), and the multigene marker, TIL-B (computed by TIMER), were consistently associated with each other across most cancer types. Importantly, both *MS4A1* and TIL-B levels are consistently prognostic across five cancer types, namely HNSCC, LUAD, CESC, LGG, and KIRP, with distinct Be2 and BK signatures (especially for *IL2*, *IL6*, and *PD-L2* expressions). In HNSCC, the favorable prognostication of high *MS4A1* and TIL-B levels identified in our study are consistent with a recent finding on the presence of high percentage of activated B-cell, antigen-presenting B-cells in HNSCC patient tumors, supportive of potential antitumor activity of TIL-B in this cancer type^30^. Moreover, Hladikova et al. demonstrated that in oropharyngeal squamous cell carcinoma, a subtype of HNSCC, high abundance of TIL-B was correlated with activated T-cell phenotype, which may favor antitumor effects^60^. In lung cancer, Bruno et al. showed that activated TIL-Bs could present antigens to CD4^+^ TIL-Ts and induce effector T-cell responses^61^, supportive of TIL-B’s antitumor activity in LUAD. In kidney cancer, cervical cancer and LGG, though some studies have demonstrated similar findings as ours^62–64^, the underline biological mechanisms have not been investigated yet.

Our findings from TCGA dataset suggest that CD20 protein level alone may not be the most reliable B-cell biomarker when assessing B-cell infiltrations in human tumors. Instead, *MS4A1*, TIL-B, or potentially *GPR18* may represent more versatile B-cell markers for prognosis in human cancers. For HNSCC, all these three potential biomarkers for TIL-B are predictive of patient outcomes, but not CD20 protein levels. In fact, Woo et al. recently stated a similar challenge for using CD20 protein detection by IHC for prostate cancer prognosis^65^, highlighting the caveats of CD20 detection by IHC. These CD20 IHC caveats include potential sampling errors for large tumors, intra- and interobserver variability in quantifying lymphocytes, the semiquantitative “eyeballing” scoring nature, lack of validated standards and staining variability, etc. Whereas the omics data have the advantages of being large scale and high throughput, they still do have worth-noting intrinsic limitations. For instance, stromal information is largely lacking in TCGA and TCPA omics data. Thus, our conclusions on survival could be further compounded by key stromal factors, such as intratumoral vessel density and inflammation status or fibrosis status of patient tumors. It will be interesting to include future data regarding stromal components, if available, for higher levels of omics analysis.

Recent studies reported on the identification of CD20-negative B-cells in colorectal, breast, and ovarian cancers. Furthermore, CD20 is expressed in B-cells from early-to-late stages, but downregulated once differentiation into plasma cells^66^. Therefore, CD20 protein detection alone may not reliably predict various TIL-Bs in human tumors. Whereas the multigene RNA-Seq based deconvolution method TIMER calculates all TIL-B subtypes includes memory B-cell, germinal center B-cell, naive B-cell, and plasma cell (CD20-negative), it is more representative of TIL-B richness in patient tumors than CD20 protein level alone. Yet, clinically, TIL-B analysis and computation can be less than ideal as it involves RNA-Seq analysis of >100 genes.

Here, we first identified a previously unreported B-cell prognostic gene, *GPR18*, whose expressions have versatile prognosticity in as many as nine cancer types, which is superior over CD20 protein and *MS4A1* mRNA. Thus, adopting *GPR18* for infiltrating B-cell assessment and cancer prognosis can be feasible in theory. Furthermore, we first uncovered *GPR18*’s potential link to cytolytic T-cell activity across pan-cancers. Future functional studies of *GPR18* in T-cell immunity should be warranted. It will be important to investigate further the prognostic value of GPR18 protein (which is lacking in the current TCPA omic data) as a biomarker for possible clinical use.

Lastly, the findings that CD20 levels are not correlated with *MS4A1* or TIL-B levels appear to support a possibly complex mode of CD20 regulation in cancer. The low rate of apparent loss-of-function mutations of *MS4A1*, including nonsense and frameshift mutations or gene fusions (<1% of TCGA pan-cancer total of 10437 samples) cannot fully account for such as major discrepancies between CD20 protein expressions and full-length *MS4A1* mRNA expressions in tumors. Though methylation could silence the expression of *MS4A1* gene, *MS4A1* gene methylation only negatively correlated with CD20 protein expressions in two cancer types (Supplementary Fig. 8). It is likely that other mechanisms may be in play to affect CD20 gene or protein expressions or detection, which are not captured by current TCGA multiomics data. One such possibility is the presence of shorter forms of CD20 protein due to *MS4A1* mRNA alternative splicing. In fact, 9 out of 13 known *MS4A1* transcripts (Supplementary Table 5) represent shorter forms of CD20 protein, which if expressed, would be undetectable by the current TCPA CD20 antibody per epitope consideration ([EP459Y], Abcam, ab78237)^67–69^.

In conclusion, quantitative levels of *GPR18*, *MS4A1*, and TIL-B are reliable biomarkers for intratumoral B-cell assessments over CD20 protein, especially for prognostic purposes in cancer. As a single gene, detection of *GPR18* mRNA could be more versatile than *MS4A1* mRNA for pan-cancer prognosis (predictive in nine vs. six cancer types, respectively). In particular, *GPR18* mRNA levels can be prognostic among HPV(+)HNSCC patients, for which CD20 protein, *MS4A1* and TIL-B levels were not prognostic at all.

## Methods

### Datasource

The RNA-Sequencing normalized TPM data of TCGA cohorts were downloaded from the Firehose (http://firebrowse.org/) in October 2019. Clinical data were extracted from cBioPortal (http://www.cbioportal.org/) in October 2019. Pan-cancer protein expression levels generated by RPPA were from TCPA (https://tcpaportal.org/tcpa/). The TCGA-independent validation breast cancer cohort data were downloaded from UCSC Xena browser with the R package UCSCXenaTools, and the GSE37745, CAARRAY lung cancer cohorts data were extracted from Kaplan–Meier plotter^42^. The *GPR18* expression data in immune cell lines were obtained from the Human Primary Cell Atlas.

### Survival analysis

Prism (3.0) was employed for Kaplan–Meier survival analyses with log-rank test. The Cox proportional hazard model was used to estimate the Hazar Ration (HR) of independent prognostic factors for overall survival (OS) with R package survival and survminer.

### TIMER and ssGSEA analyses

To compute the abundance of immune cell infiltration in HNSCC¸ TIMER bioinformatics approach was employed using the TCGA RNA-Seq data^25^. For pan-cancer analyses, the TIL values for ACC, BLCA, BRCA, CESC, COAD, CHOL, DLBC, ESCA, GBM, kidney chromophobe, KIRC, KIRP, LGG, LIHC, LUAD, LUSC, OV, PAAD, prostate adenocarcinoma, rectum adenocarcinoma, SARC, SKCM, STAD, TGCT, THCA, THYM, UCEC, and UVM were downloaded from TIMER website directly (https://cistrome.shinyapps.io/timer/).

ssGSEA was used to compute the infiltration level of 24 immune cell types, which was adopted from previous study with R package gsva^33^.

### B-cell prognostic signature gene analysis

For B-cell prognostic signature gene analysis, the 114 B-cell marker genes were selected from TIMER TCGA-HNSCC 449 immune gene set, which contains the genes positively correlated with immune cell level and negatively correlated with tumor purity. Only the immune signature genes markedly expressed in B-cells (average TPM > 100 in B-cell-related cell lines) were filtered as B-cell marker genes. Univariate Cox-regression was performed in five TIL-B and *MS4A1*-prognostic cancer types. Only the genes that were significantly (*P* < 0.05) associated with OS in all five cancer types would be considered as B-cell prognostic signature genes (Fig. 3a).

### Heatmaps

To compare the immune profiles between TIL-B high and low tumors (median cutoff) in the five TIL-B and *MS4A1*-prognostic cancer types, the ssGSEA scores of 24 immune cell types were generated to calculate the score differences and statistically significance values. The score differences were shown in the heatmap with statistically significant results in color and non-significant ones in gray. To compare the functional signature of TIL-B between TIL-B high and low tumors in HNSCC, LUAD, CESC, LGG, and KIRP (median cutoff), the TPMs of Be1, Be2, Breg, and BK marker genes were extracted and transformed to log2(TPM + 1) to calculate the fold change and statistical significance. Fold changes were presented in heatmap with statistically significant results in color and the non-significant ones in gray. The correlation heatmaps were generated based on the Pearson correlations and unsupervised clustering. The complete linkage method was used for clustering in each heatmap. The heatmaps were generated with R package pheatmap (version 1.0.12) and TBtools (version 0.6695).

### Statistics and reproducibility

Pearson correlation coefficiency was used to evaluate the relationship between two datasets. |*R*| > 0.2 and *P* < 0.05 was defined as significant correlation. Unpaired Student’s *t* test or log-rank test was used to compare the difference between two groups. *P* < 0.05 is considered as statistically significant. * is for 0.01 < *P* < 0.05, ** is for 0.001 < *P* < 0.01, *** is for 0.0001 < *P* < 0.001, and **** is for *P* < 0.0001. The detailed *N* numbers of respective pan-cancer analyses in this study are presented in Supplementary Table 2.

### Reporting summary

Further information on research design is available in the Nature Research Reporting Summary linked to this article.

## Supplementary information

Supplementary Information

Reporting Summary

## Data Availability

All relevant data are available from the authors upon request. Corresponding author and first author are responsible for such requests. The detailed *N* numbers of respective pan-cancer analyses in this study are presented in Supplementary Table 2. All the source data underlying the graphs and charts are available on Figshare^70^.

## Code availability

R script used in this publication could be accessed via https://gocuhk-my.sharepoint.com/:f:/g/personal/yuchenliu_cuhk_edu_hk/Ek-Q2uRjLIFEkyiqDKmzAFMB6pT-ciRYC4t4UX7kDKyk1A.
