# Supplementary material for: Omics-wide quantitative B-cell infiltration analyses identify GPR18 for human cancer prognosis with superiority over CD20
Source: Commun Biol. 2020 May 12;3:234. doi: 10.1038/s42003-020-0964-7 (PMC7217858; doi:10.1038/s42003-020-0964-7)
Supplement: Supplementary file 1 — Supplementary Information [file 42003_2020_964_MOESM1_ESM.pdf]

Supplementary Information:

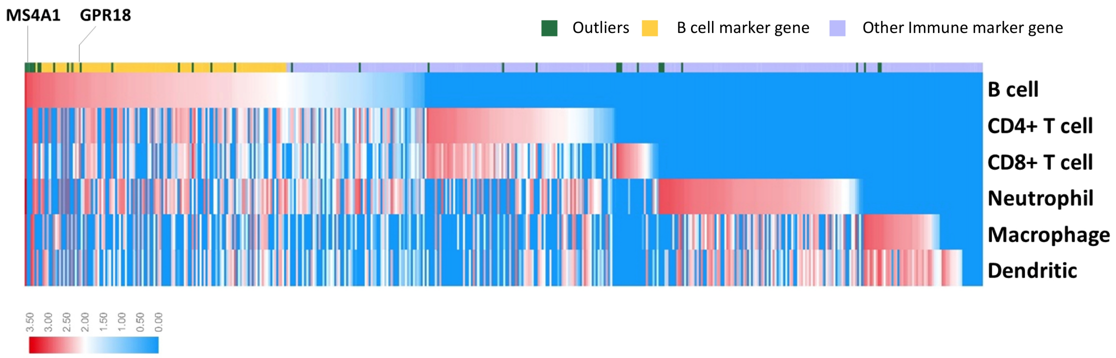

Supplementary Figure 1. Expression patterns of 477 TCGA-HNSCC immune marker genes (with outliers) in six immune cell lines. *MS4A1* and *GPR18* are labeled. (B cell, N=24. $CD4^{+}$ T cell, N=27. $CD8^{+}$ T cell, N=28. Dendritic, N=88. Macrophage, N=15. Neutrophil, N=18.).

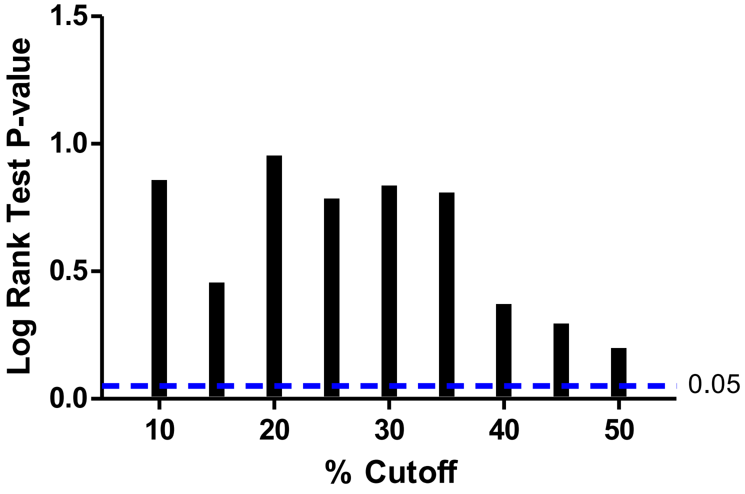

Supplementary Figure 2. CD20 protein levels are not prognostic in HNSCC. Statistical screen for significance (P values of Log-rank test for overall survival) for patients based on their quantitative CD20 protein expression levels (from top10% vs bottom10%, to top50% vs bottom50%).

	beta	SE	HR (95% CI for HR)	p.value
HNSCC	0.194	0.338	1.21 (0.626-2.35)	0.567
LUAD	0.0248	0.338	1.03 (0.528-1.99)	0.942
CESC	-1.25	0.719	0.285 (0.0696-1.17)	0.0811
LGG	-0.936	0.528	0.392 (0.139-1.1)	0.0763
KIRP	-0.191	1.09	0.826 (0.0966-7.06)	0.861
ACC	-4.21	2.74	0.0149 (6.93e-05-3.18)	0.124
BLCA	-0.958	0.536	0.384 (0.134-1.1)	0.0739
BRCA	-0.849	0.494	0.428 (0.163-1.13)	0.0859
CHOL	0.0367	1.51	1.04 (0.0535-20.1)	0.981
COAD	-0.126	0.695	0.882 (0.226-3.44)	0.856
DLBC	-0.825	0.555	0.438 (0.148-1.3)	0.137
ESCA	0.802	0.48	2.23 (0.871-5.71)	0.0947
GBM	0.0689	0.348	1.07 (0.541-2.12)	0.843
KICH	0.171	2.14	1.19 (0.0181-78)	0.936
KIRC	-0.209	0.327	0.811 (0.427-1.54)	0.522
LIHC	0.142	0.61	1.15 (0.349-3.81)	0.815
LUSC	0.378	0.547	1.46 (0.499-4.26)	0.49
OV	-0.867	0.481	0.42 (0.164-1.08)	0.0713
PAAD	1.65	0.602	5.22 (1.6-17)	**0.00605**
PRAD	0.632	1.26	1.88 (0.158-22.4)	0.617
READ	0.492	0.673	1.64 (0.437-6.12)	0.464
SARC	-0.337	0.317	0.714 (0.383-1.33)	0.288
SKCM	-0.561	0.703	0.571 (0.144-2.27)	0.425
STAD	0.319	0.12	1.38 (1.09-1.74)	**0.00798**
TGCT	-0.903	2.12	0.405 (0.00633-26)	0.67
THCA	0.75	0.462	2.12 (0.856-5.24)	0.105
THYM	-0.744	1.27	0.475 (0.0392-5.76)	0.559
UCEC	-0.438	0.519	0.645 (0.233-1.79)	0.399

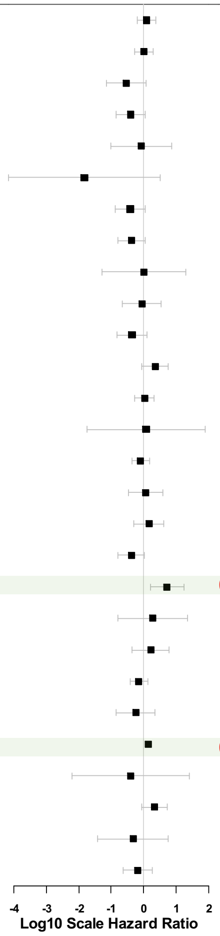

Supplementary Figure 3. Forestplot showing the univariate Cox regression analyses for quantitative CD20 protein levels across cancer types. The N number for each cohort are shown in supplementary Table 2.

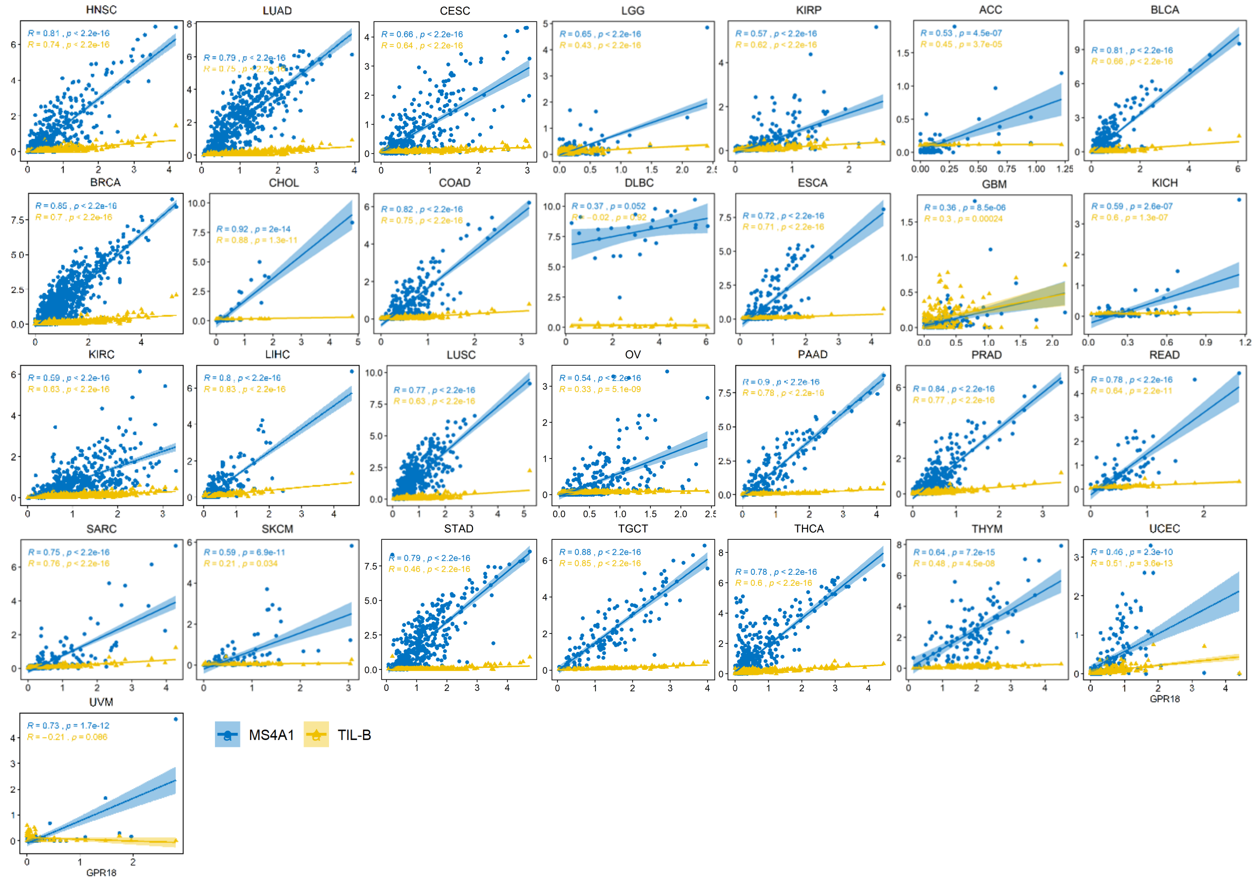

Supplementary Figure 4. Pearson's correlations between *GPR18* mRNA and *MS4A1* mRNA/TIL-B levels across 29 cancer types. The N numbers for each plot are shown in supplementary Table 2.

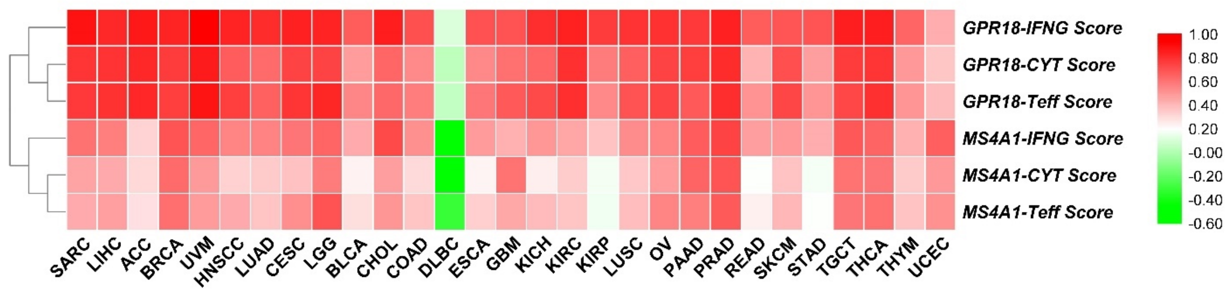

Supplementary Figure 5. Pearson's correlations between *GPR18/MS4A1* and three T cell immunoreactive signature scores (CYT, Teff and IFNG) across 29 cancer types. The N numbers are shown in supplementary Table 2.

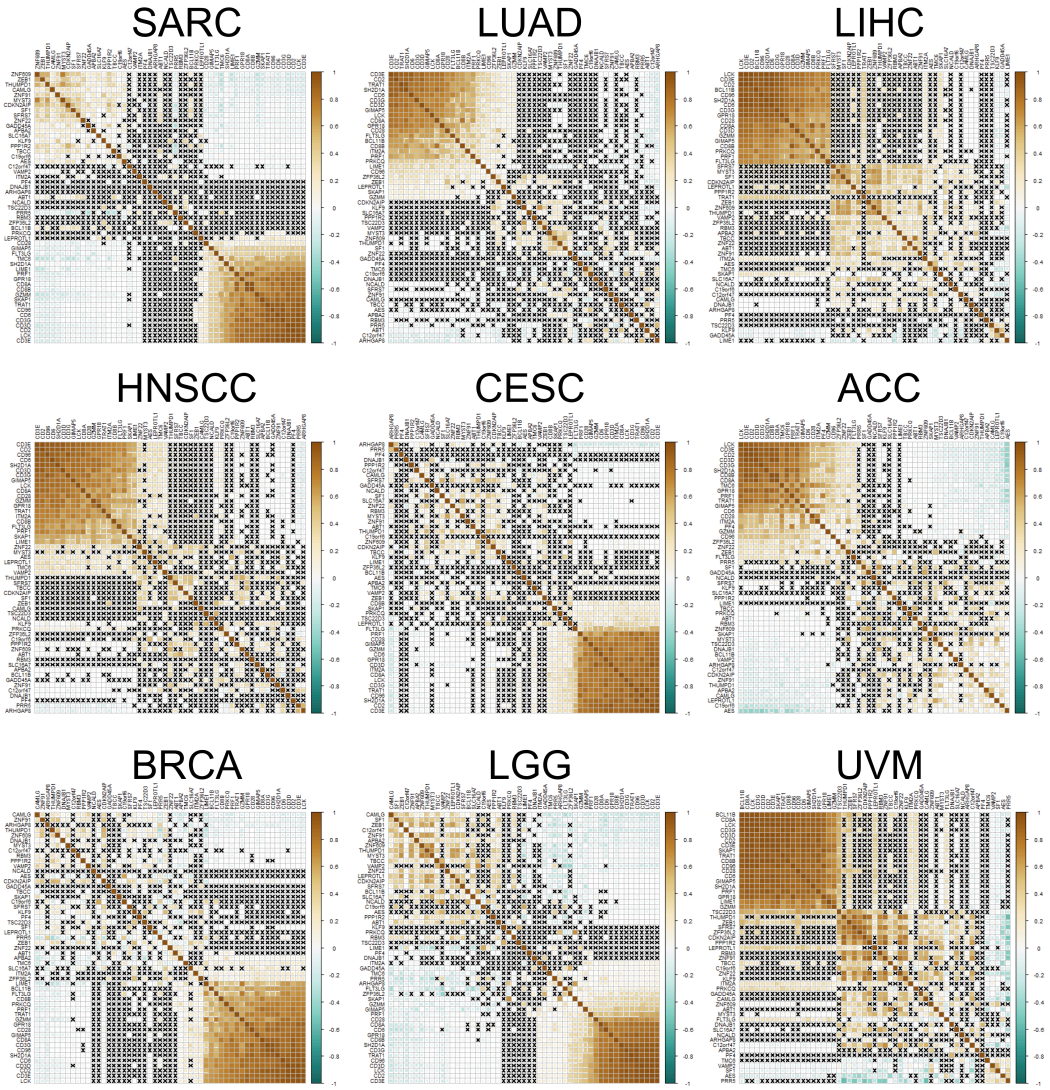

Supplementary Figure 6. Correlation heatmap between *GPR18* and cytotocic T cell marker genes in 9 *GPR18* prognostic cancer types. The N numbers are shown in supplementary Table 2.

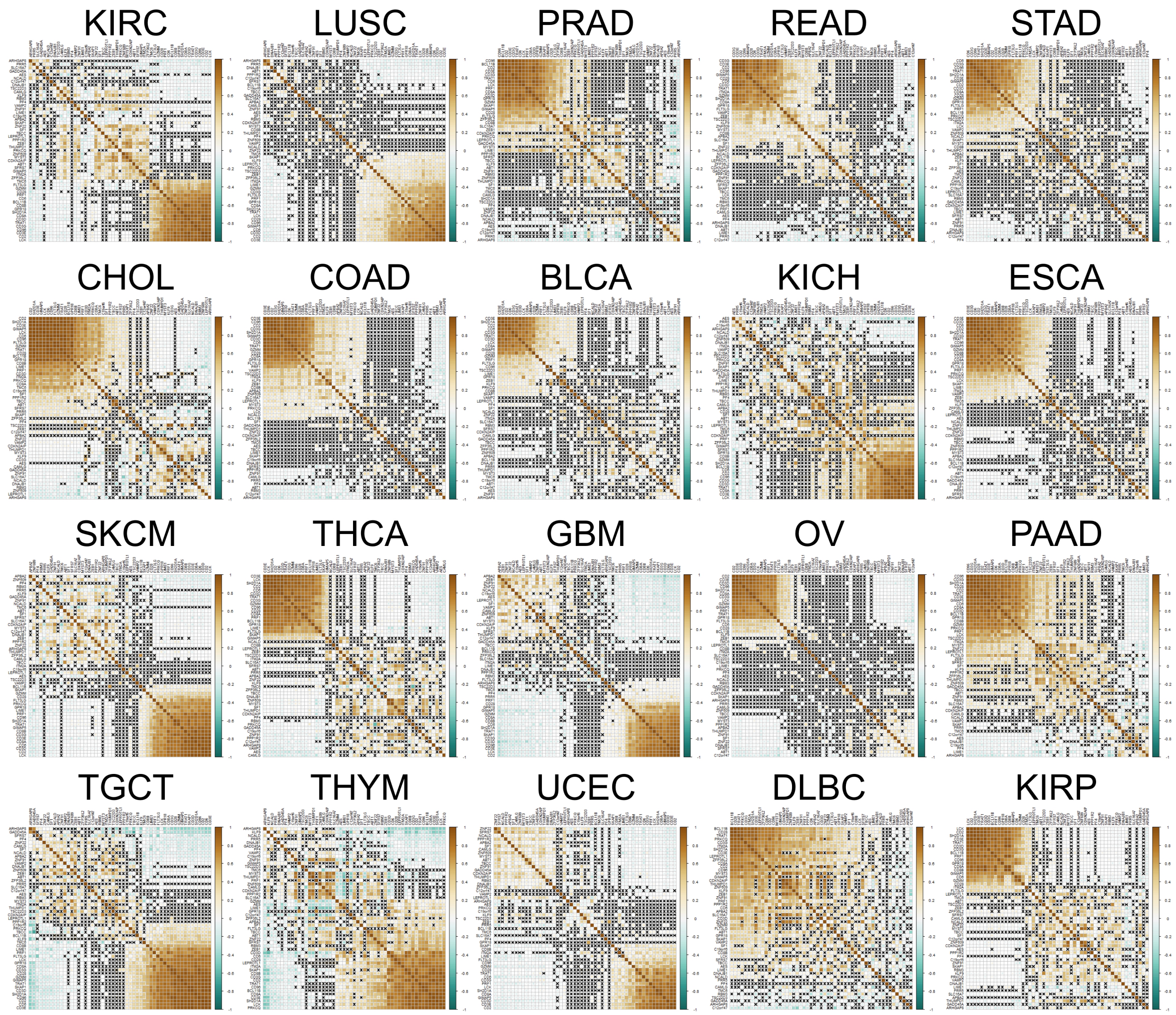

Supplementary Figure 7. Correlation heatmap between *GPR18* and cytotocic T cell marker genes in 20 *GPR18* not prognostic cancer types. The N numbers are shown in supplementary Table 2.

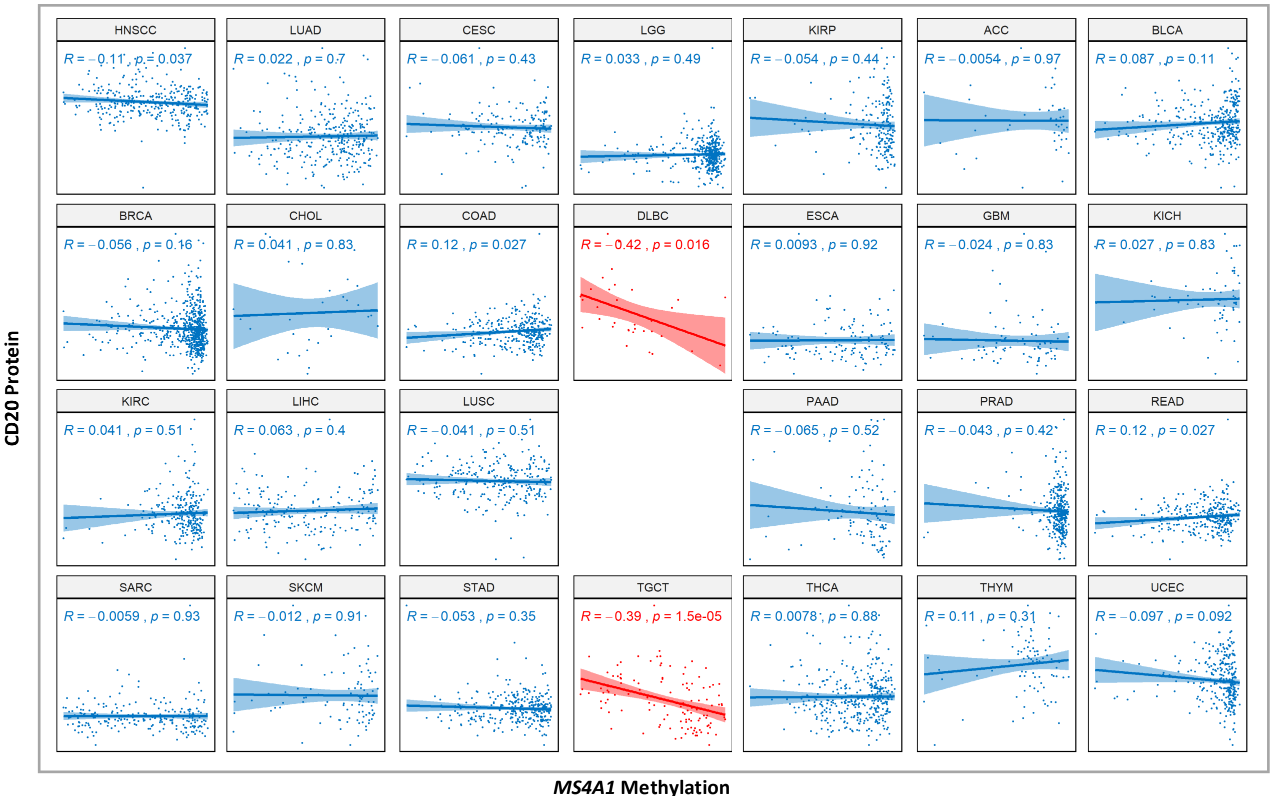

Supplementary Figure 8. Pearson's correlations between *MS4A1* methylation CD20 protein levels across pan-cancers. The methylation data for UVM are not available. The N numbers are shown in supplementary Table 2.

Supplementary Table 1. TCGA-HNSCC immune marker genes.

ACAP1	CD19	CPVL	FASLG	GPSM3	ITGAX	MPEG1	PTGER2	SPIB
ACP5	CD1B	CR1	FBXO6	GZMA	ITGB2	MS4A6A	PTPN22	SPOCD1
ADAP2	CD1C	CRTAM	FCGR2A	GZMB	ITGB7	MS4A7	PTPN7	ST8SIA4
ADCY7	CD1D	CSF1R	FCGR2B	GZMH	ITK	MX1	PTPRCAP	STAB1
AIF1	CD1E	CSF1	FCGR2C	GZMK	JAK3	MX2	PTPRC	STAP1
AIM2	CD209	CSF2RA	FCN1	GZMM	KBTBD8	MYO1F	PTPRE	STAT1
AKNA	CD244	CSF2RB	FCRL1	HAVCR2	KCNK6	MYO1G	PYHIN1	STAT4
ALOX5	CD247	CSF3R	FCRL2	HCK	KCTD12	NAGK	QPCT	STK10
AMICA1	CD274	CST7	FCRL3	HCLS1	KIAA0125	NAIP	RAB37	STX11
AMPD1	CD27	CTLA4	FCRL5	HCST	KIR2DL4	NCF2	RAB8B	TAGAP
APBB1IP	CD2	CTSL1	FCRLA	HK3	KLHL6	NCF4	RASAL3	TAP1
APOBEC3A	CD300A	CTSW	FERMT3	HLA-DOB	KLRB1	NCKAP1L	RASGRP1	TAP2
ARHGAP15	CD37	CTSZ	FFAR2	HLA-DQA1	KLRD1	NCR3	RASGRP2	TBC1D10C
ARHGAP18	CD3D	CX3CR1	FGL2	HLA-DQB1	KLRF1	NFAM1	RASGRP3	TBX21
ARHGAP25	CD3E	CXCL10	FGR	HLX	KLRG1	NFE2	RASGRP4	TBXAS1
ARHGAP30	CD3G	CXCL11	FLI1	HMOX1	KLRK1	NKG7	RASSF2	TFEC
ARHGAP9	CD53	CXCL9	FLVCR2	HVCN1	KYNU	NLRC3	RASSF4	TIFAB
BATF3	CD5	CXCR1	FMNL1	ICAM2	LACTB	NLRC5	RCSD1	TIGIT
BATF	CD69	CXCR6	FPR1	ICAM3	LAG3	NLRP12	RFTN1	TIMP2
BCL2A1	CD6	CYBB	FPR2	ICOS	LAT2	NR4A3	RGL4	TLR10
BIN2	CD79A	CYLD	FPR3	IDO1	LCK	NRP1	RGS18	TLR4
BLK	CD79B	CYSLTR1	FXYD5	IFI35	LCP1	NT5C3	RGS1	TLR8
BLNK	CD7	CYTH4	FYB	IFI44L	LCP2	OAS1	RHOG	TMEM149
BMP2K	CD80	CYTIP	FYN	IFI44	LILRA1	OAS2	RHOH	TMEM156
BTLA	CD84	DDX60L	GBP1	IFI6	LILRA2	OAS3	RNASE2	TMIGD2
BTN3A2	CD86	DERL3	GBP5	IFIH1	LILRA6	OBFC2A	RNASE6	TNFAIP6
C10orf54	CD8B	DNAJC5B	GGTA1	IFIT1	LILRB1	OSM	RNF213	TNFRSF17
C11orf21	CD96	DOCK10	GIMAP2	IFIT3	LILRB2	P2RY13	RSAD2	TNFRSF1B
C13orf18	CD97	DOCK2	GIMAP4	IFNG	LILRB3	P2RY8	S1PR4	TNFRSF4
C13orf31	CDA	DOCK8	GIMAP5	IGSF6	LPXN	PARP9	SAMD3	TNFRSF9
C1orf162	CECR1	DOK3	GIMAP6	IKZF1	LRMP	PARVG	SAMD9L	TNFSF13B
C1orf38	CELF2	DPEP2	GIMAP7	IL10RA	LRRN3	PATL2	SAMD9	TNFSF8
C5AR1	CFLAR	DPYD	GJB2	IL12B	LSP1	PDCD1LG2	SAMHD1	TNIP3
C5orf20	CFP	DUSP4	GLIPR1	IL13RA1	LST1	PDE4B	SAMSN1	TRAF1
C5orf56	CHI3L1	EBI3	GLIPR2	IL16	LTA	PIK3CD	SASH3	TRAF3IP3
CASP1	CHST11	ELL2	GLT1D1	IL18RAP	LTB	PIK3R5	SELPLG	TRAT1
CCDC109B	CISH	EMR2	GMFG	IL24	LY96	PILRA	SEPX1	TREM2
CCL13	CLC	EMR3	GNG2	IL2RA	LY9	PIM2	SERPINB9	TRIM69
CCL17	CLEC10A	ENTPD1	GNG7	IL2RB	MAN1A1	PLA2G7	SH2D2A	TRPV2
CCL19	CLEC4A	EOMES	GNLY	IL2RG	MAP4K1	PLEK	SIGLEC1	TYMP
CCL22	CLEC4E	EREG	GPR114	IL32	MCOLN2	PLXNC1	SLAMF1	VNN2
CCL24	CLEC5A	EVI2A	GPR132	IL3RA	MEFV	PNOC	SLAMF7	WAS
CCL4	CLEC7A	EVI2B	GPR157	IL4R	MEI1	POU2F2	SLAMF8	WIPF1
CCL8	CLECL1	FAIM3	GPR15	INPP5D	MFNG	PRAM1	SLA	XAF1
CCR1	CLIC3	FAM113B	GPR171	IRF4	MGC29506	PRDM1	SLC15A3	ZAP70
CCR2	CMPK2	FAM129C	GPR183	IRF7	MIR155HG	PREX1	SLC31A2	ZBP1
CCR3	CORO1A	FAM26F	GPR18	IRF8	MMP1	PRF1	SMAP2	ZEB2
CCR5	COTL1	FAM46C	GPR65	ISG20	MMP25	PRKCQ	SP110	ZNF467
CCR7	CPM	FAM49A	GPR68	ITGA4	MMP9	PROK2	SP140L	ZNFX1
CCRL2	CPNE5	FAM65B	GPR84	ITGAL	MNDA	PTGDR	SPI1	

Supplementary Table 2. N numbers of analyses

Cancer Type	Full Name	Fig 1c	Fig 1d, SFig 3	Fig 1d, Fig 3b	Fig 1d, Fig 3b	Fig 1e-1g	SFig 4, SFig 5	SFig6, SFig7	SFig 8
HNSCC	head and neck squamous cell carcinoma	346	344	518	512	335	514	520	520
LUAD	lung adenocarcinoma	362	354	506	502	347	511	515	454
CESC	cervical squamous cell carcinoma and endocervical adenocarcinoma	171	171	304	304	169	304	304	304
LGG	lower grade glioma	427	425	514	514	425	515	516	516
KIRP	kidney papillary cell carcinoma	208	206	289	289	205	290	290	274
ACC	adrenocortical carcinoma	46	46	79	79	46	79	79	79
BLCA	bladder urothelial carcinoma.	344	342	406	405	337	407	408	408
BRCA	breast invasive carcinoma	874	872	1091	1089	866	1091	1093	780
CHOL	cholangiocarcinoma	30	28	34	34	28	36	36	36
COAD	colon adenocarcinoma	357	350	282	454	231	283	285	277
DLBC	diffuse large B-cell lymphoma	33	33	48	28	19	28	48	48
ESCA	esophageal carcinoma	126	126	184	184	125	184	184	184
GBM	glioblastoma multiforme	205	203	151	523	66	149	153	51
KICH	kidney chromophore	63	62	65	65	62	66	66	66
KIRC	kidney renal clear cell carcinoma	445	445	533	533	442	533	533	318
LIHC	liber hepatocellular carcinoma	184	184	370	370	181	371	371	371
LUSC	lung squamous cell carcinoma	325	320	495	495	317	501	501	370
OV	ovarian serous cystadenocarcinoma	411	406	302	563	226	294	303	NA
PAAD	pancreatic adenocarcinoma	105	105	178	178	98	178	178	178
PRAD	prostate adenocarcinoma	351	351	497	497	350	497	497	497
READ	rectum adenocarcinoma	130	129	89	160	72	91	94	92
SARC	sarcoma	221	221	259	257	218	257	259	259
SKCM	skin cutaneous melanoma	353	96	103	103	90	102	103	103
STAD	stomach adenocarcinoma	392	349	407	401	318	404	415	372
TGCT	testicular germ cell tumor	118	104	134	134	104	134	150	150
THCA	thyroid carcinoma	372	372	501	501	366	501	501	501
THYM	thymoma	90	89	119	118	86	119	120	120
UCEC	uterine corpus endometrial carcinoma	404	403	175	544	102	176	176	172
UVM	uveal melanoma	12	NA	68	68	10	80	80	NA

Supplementary Table 3. B cell gene list

ACAP1	IKZF1	SP140L
AIM2	IL16	SPIB
ALOX5	IL4R	STAP1
APBB1IP	INPP5D	STAT1
ARHGAP25	IRF7	TAGAP
BLK	IRF8	TAP1
BLNK	ISG20	TAP2
BMP2K	JAK3	TBC1D10C
BTN3A2	KBTBD8	TLR10
C13orf18	KIAA0125	TNFRSF17
CCDC109B	KLHL6	TYMP
CD19	LAT2	VNN2
CD27	LCK	WAS
CD37	LCP1	WIPF1
CD53	LPXN	
CD79A	LRMP	
CD79B	LTB	
CLECL1	LY9	
CORO1A	MAP4K1	
COTL1	MCOLN2	
CPNE5	MFNG	
CYBB	MGC29506	
CYTIP	MMP1	
DOCK2	MX1	
DOCK8	NAGK	
DOK3	NCF4	
ENTPD1	NLRC3	
EVI2B	NT5C3	
FAM113B	P2RY8	
FAM129C	PARVG	
FAM26F	PDE4B	
FAM46C	PIK3CD	
FAM65B	PNOC	
FCRL1	POU2F2	
FCRL2	PTPN7	
FCRL3	PTPRCAP	
FCRL5	PTPRC	
FCRLA	RAB8B	
FERMT3	RASGRP1	
FLI1	RASGRP3	
GJB2	RASSF2	
GMFG	RCSD1	
GNG2	RFTN1	
GNG7	RGS1	
GPR114	RHOG	
GPR18	RHOH	
HCLS1	SASH3	
HVCN1	SERPINB9	
ICAM3	SMAP2	
IFI6	SP110	

Supplementary Table 4. Genes clustered with *MS4A1* across cancer types

Genes	ACC	CESC	HNSCC	LGG	LUAD	SARC	UVM	Total
GPR18	1	1	1	1	1	1	1	**7**
ACAP1		1	1	1	1	1	1	**6**
LCK	1	1		1	1	1		**5**
PTPRCA	1		1		1	1	1	**5**
TBC1D1	1		1		1	1	1	**5**
CORO1A		1	1		1		1	**4**
LTB	1		1	1			1	**4**
MAP4K1	1		1		1	1		**4**
PTPN7		1			1	1	1	**4**
CD27	1					1		**2**
CD37		1	1					**2**
FCRL3			1			1		**2**
LY9	1					1		**2**
NLRC3		1			1			**2**
SASH3		1					1	**2**
BLNK							1	**1**
CLECL1							1	**1**
CYTIP							1	**1**
EVI2B							1	**1**
FAM26F	1							**1**
GPR114					1			**1**
IKZF1							1	**1**
IL16		1						**1**
JAK3					1			**1**
LCP1							1	**1**
LRMP							1	**1**
P2RY8							1	**1**
POU2F2							1	**1**
RHOH							1	**1**
TBC1D10				1				**1**
WAS		1						**1**

Supplemantary Table 5. Summary of *MS4A1* mRNA alterative splicing.

Name	Transcript ID	Protein	CCDS	UniProt	Length of CDS	Reference
MS4A1-212	ENST00000534668.5	297aa	CCDS31570	A0A024R507 P11836	894	Ensembl
MS4A1-202	ENST00000389939.2	297aa	CCDS31570	A0A024R507 P11836	894	Ensembl
MS4A1-201	ENST00000345732.8	297aa	CCDS31570	A0A024R507 P11836	894	Ensembl
MS4A1-207	ENST00000532073.5	284aa	-	E9PKH8	-	Ensembl
MS4A1-205	ENST00000528313.1	130aa	-	P11836	-	Ensembl
MS4A1-210	ENST00000533306.5	123aa	-	E9PPL6	-	Ensembl
MS4A1-209	ENST00000532491.5	6aa	-	-	-	Ensembl
MS4A1-203	ENST00000524807.5	7aa	-	A0A1Y8EK32	-	Ensembl
D393-CD20	-	131aa	-	-	393	Gamonet et al. and Herny et al.
D657-CD20	-	219aa	-	-	657	Gamonet et al.
D618-CD20	-	206aa	-	-	618	Gamonet et al.
D480-CD20	-	160aa	-	-	480	Gamonet et al.
D177-CD20	-	59aa	-	-	177	Gamonet et al.
