# Supplementary material for: Omics-wide quantitative B-cell infiltration analyses identify GPR18 for human cancer prognosis with superiority over CD20
Source: Commun Biol. 2020 May 12;3:234. doi: 10.1038/s42003-020-0964-7 (PMC7217858; doi:10.1038/s42003-020-0964-7)
Supplement: Supplementary file 2 — Reporting Summary [file 42003_2020_964_MOESM2_ESM.pdf]

nature research

Corresponding author(s): Vivian Wai Yan Lui

Last updated by author(s): Apr 6, 2020

Reporting Summary

Nature Research wishes to improve the reproducibility of the work that we publish. This form provides structure for consistency and transparency in reporting. For further information on Nature Research policies, see Authors & Referees and the Editorial Policy Checklist.

Statistics

For all statistical analyses, confirm that the following items are present in the figure legend, table legend, main text, or Methods section.

n/a	Confirmed	
☐	☒	The exact sample size (n) for each experimental group/condition, given as a discrete number and unit of measurement
☒	☐	A statement on whether measurements were taken from distinct samples or whether the same sample was measured repeatedly
☐	☒	The statistical test(s) used AND whether they are one- or two-sided<br>*Only common tests should be described solely by name; describe more complex techniques in the Methods section.*
☒	☐	A description of all covariates tested
☒	☐	A description of any assumptions or corrections, such as tests of normality and adjustment for multiple comparisons
☐	☒	A full description of the statistical parameters including central tendency (e.g. means) or other basic estimates (e.g. regression coefficient) AND variation (e.g. standard deviation) or associated estimates of uncertainty (e.g. confidence intervals)
☐	☒	For null hypothesis testing, the test statistic (e.g. F, t, r) with confidence intervals, effect sizes, degrees of freedom and P value noted<br>*Give P values as exact values whenever suitable.*
☒	☐	For Bayesian analysis, information on the choice of priors and Markov chain Monte Carlo settings
☒	☐	For hierarchical and complex designs, identification of the appropriate level for tests and full reporting of outcomes
☐	☒	Estimates of effect sizes (e.g. Cohen's d, Pearson's r), indicating how they were calculated

Our web collection on statistics for biologists contains articles on many of the points above.

Software and code

Policy information about availability of computer code

Data collection	R version 2.6.1, R package UCSCXenaTools 1.2.9 were used to collect data in this manuscript.
Data analysis	R version 2.6.1, R package survival 2.44.1.1, survminer 0.4.6, gsva 1.34.0, pheatmap 1.0.12, Prism 3.0 and TBtools 0.6695 were used to analyze data in this manuscript.

For manuscripts utilizing custom algorithms or software that are central to the research but not yet described in published literature, software must be made available to editors/reviewers. We strongly encourage code deposition in a community repository (e.g. GitHub). See the Nature Research guidelines for submitting code & software for further information.

Data

Policy information about availability of data

All manuscripts must include a data availability statement. This statement should provide the following information, where applicable:

- Accession codes, unique identifiers, or web links for publicly available datasets
- A list of figures that have associated raw data
- A description of any restrictions on data availability

The RNA-Seq normalized TPM (Transcript Per Million) data of TCGA cohorts was downloaded from Firehose (http://firebrowse.org/) in Oct-2019. Clinical data was extracted from cbioportal (http://www.cbioportal.org/) in Oct-201959. Pan-cancer protein expression levels generated by RPPA were provided by TCPA (https://tcpaportal.org/tcpa/). The TCGA independent validation breast cancer cohort data was downloaded from UCSC Xena browser with the R package UCSCXenaTools and GSE37745, CAARRAY lung cancer cohorts data was extracted from Kaplan-Meier Plotter. The GPR18 expression data in immune cell lines was got from the Human Primary Cell Atlas (HPCA).

Field-specific reporting

Please select the one below that is the best fit for your research. If you are not sure, read the appropriate sections before making your selection.

☒ Life sciences ☐ Behavioural & social sciences ☐ Ecological, evolutionary & environmental sciences

For a reference copy of the document with all sections, see nature.com/documents/nr-reporting-summary-flat.pdf

Life sciences study design

All studies must disclose on these points even when the disclosure is negative.

Sample size	Sample size was determined by the number of primary tumors in each cohorts
Data exclusions	Only the primary tumors with RNA-Seq/RPPA and clinical data were included in this study.
Replication	Not applicable.
Randomization	Not applicable.
Blinding	Not applicalbe

Reporting for specific materials, systems and methods

We require information from authors about some types of materials, experimental systems and methods used in many studies. Here, indicate whether each material, system or method listed is relevant to your study. If you are not sure if a list item applies to your research, read the appropriate section before selecting a response.

Materials & experimental systems

n/a	Involved in the study	
☒	☐	Antibodies
☒	☐	Eukaryotic cell lines
☒	☐	Palaeontology
☒	☐	Animals and other organisms
☒	☐	Human research participants
☒	☐	Clinical data

Methods

n/a	Involved in the study	
☒	☐	ChIP-seq
☒	☐	Flow cytometry
☒	☐	MRI-based neuroimaging
